# Profiling of cool-season forage arabinoxylans *via* a validated HPAEC-PAD method

**DOI:** 10.3389/fpls.2023.1116995

**Published:** 2023-03-13

**Authors:** Glenna E. Joyce, Isabelle A. Kagan, Michael D. Flythe, Brittany E. Davis, Rachel R. Schendel

**Affiliations:** ^1^ Department of Animal and Food Sciences, University of Kentucky, Lexington, KY, United States; ^2^ Forage-Animal Production Research Unit, U.S. Department of Agriculture, Agricultural Research Service (USDA-ARS), Lexington, KY, United States

**Keywords:** hemicellulose, endoxylanase, plant cell wall, pasture grass, hydroxycinnamic acid

## Abstract

Cool-season pasture grasses contain arabinoxylans (AX) as their major cell wall hemicellulosic polysaccharide. AX structural differences may influence enzymatic degradability, but this relationship has not been fully explored in the AX from the vegetative tissues of cool-season forages, primarily because only limited AX structural characterization has been performed in pasture grasses. Structural profiling of forage AX is a necessary foundation for future work assessing enzymatic degradability and may also be useful for assessing forage quality and suitability for ruminant feed. The main objective of this study was to optimize and validate a high-performance anion-exchange chromatography with pulsed amperometric detection (HPAEC-PAD) method for the simultaneous quantification of 10 endoxylanase-released xylooligosaccharides (XOS) and arabinoxylan oligosaccharides (AXOS) in cool-season forage cell wall material. The following analytical parameters were determined or optimized: chromatographic separation and retention time (RT), internal standard suitability, working concentration range (CR), limit of detection (LOD), limit of quantification (LOQ), relative response factor (RRF), and quadratic calibration curves. The developed method was used to profile the AX structure of four cool-season grasses commonly grown in pastures (timothy, *Phleum pratense* L.; perennial ryegrass, *Lolium perenne* L.; tall fescue, *Schedonorus arundinaceus* (Schreb.) Dumort.; and Kentucky bluegrass, *Poa pratensis* L.). In addition, the cell wall monosaccharide and ester-linked hydroxycinnamic acid contents were determined for each grass. The developed method revealed unique structural aspects of the AX structure of these forage grass samples that complemented the results of the cell wall monosaccharide analysis. For example, xylotriose, representing an unsubstituted portion of the AX polysaccharide backbone, was the most abundantly-released oligosaccharide in all the species. Perennial rye samples tended to have greater amounts of released oligosaccharides compared to the other species. This method is ideally suited to monitor structural changes of AX in forages as a result of plant breeding, pasture management, and fermentation of plant material.

## 1 Introduction

Cool-season forage grasses are commonly incorporated into grazing systems for livestock in temperate climates. As monocots, the cell walls of grasses are distinguished by substantial amounts of feruloylated arabinoxylans (AX), a complex hemicellulosic polysaccharide (Vogel, 2008). The structure of AX is based around a semi-flexible backbone of β-1→4-linked xylopyranose units substituted with a variety of branching units. Monomeric arabinofuranose is the most abundant branching unit, but other backbone substituents are also present, including short oligosaccharide side-chains originating at an arabinofuranose unit, glucuronic acid, and acetyl groups (Albersheim et al., 2010). Structural complexity is further increased by the presence of substantial amounts of ester-linked phenolic acids, which acylate the *O*-5 position of some of the monomeric arabinofuranose units and the arabinose-containing oligosaccharide side-chains (Schendel et al., 2016).

AX fill a crucial structural role in the grass cell wall by crosslinking the major cell wall polymers. In the case of cellulose, AX tether cellulose microfibrils together by forming hydrogen-bonding regions between multiple microfibrils and by being trapped in the microfibril as it crystallizes (Scheller and Ulvskov, 2010). Importantly, differences in AX structure affect this behavior, specifically the extent and pattern of backbone substitution, with denser substitution patterns limiting hydrogen-bonding interactions with cellulose (Saulnier et al., 2012). AX also build AX-AX and AX-lignin crosslinkages *via* free-radical-induced oxidative coupling of ferulic acid (Ralph et al., 1994; Ralph et al., 1995; Saulnier et al., 1999).

Besides influencing the structural stability of the plant cell wall, the pattern and extent of AX backbone substitution also shift enzymatic degradability of the cell wall polysaccharides. AX are depolymerized by *endo-*1,4-β-xylanases and various accessory enzymes: β-xylosidases, α-L-arabinofuranosidases, α-glucuronidases, feruloyl esterases, acetylxylan esterases, and α-galactosidases (Crepin et al., 2004; Pollet et al., 2010; Biely et al., 2014; Lagaert et al., 2014). The enzymatic degradability of AX is influenced by many structural factors, including the degree of substitution along the xylan backbone, presence of oligosaccharide side-chains, *O*–5-feruloylation of arabinofuranose backbone substituents, and ferulate-governed crosslinking of xylan strands (Grabber et al., 1998; Beaugrand et al., 2004; Jung et al., 2012; Neumüller et al., 2014; Snelders et al., 2014).

The AX structures of the grain tissue of prominent cereal crops have been studied in detail due to interest in their human health benefits (Andersson et al., 2008; Nyström et al., 2008; Shewry et al., 2008) and role in baking and brewing quality (Biliaderis et al., 1995; Courtin and Delcour, 2002; Dervilly et al., 2002; Cyran et al., 2003; Kosik et al., 2017). However, much less is known about the AX structures of non-grain, vegetative tissues, with a few exceptions such as sugarcane and bamboo (Azuma et al., 1990; Ishii, 1991) and materials targeted for second generation biofuel production, such as barley straw and alkali-pretreated switchgrass (Höije et al., 2005; Mazumder and York, 2010; Bowman et al., 2012; Bowman et al., 2015; Tryfona et al., 2019). The knowledge gap in regards to the AX structure from the natural (non-pretreated) vegetative tissue of cool-season forage species is especially wide, despite the fact that these plants make sizeable, profitable contributions to the diets of important production animals like beef and dairy cattle (Lardner et al., 2013; Ritz et al., 2021). AX are fermented by the rumen bacteria (Russell and Rychlik, 2001; Dodd et al., 2011), but the absence of detailed structural information about AX from vegetative tissues in cool season forage species means that questions about how forage AX structure directs rumen fermentation patterns and generates downstream health and performance consequences in the host remain unanswered. In this study, we report the validation of a quantitative screening method for forage AX based on digestion with endoxylanase and separation and quantification of the released oligosaccharides using high-performance anion-exchange chromatography with pulsed amperometric detection (HPAEC-PAD). We also compare the monosaccharide composition of the cell wall polysaccharides and the ester-linked phenolic acid profile of several common cool-season forages.

## 2 Materials and methods

### 2.1 Plant materials, enzymes, chemicals, and oligosaccharide standard compounds

#### 2.1.1 Plant materials

Grass samples were collected in April 2019 from forage plots managed at the University of Kentucky’s Spindletop Farm research station (3250 Iron Works Pike, Lexington, Kentucky). Timothy (*Phleum pratense* L., cultivar ‘Clair’) samples were from a plot established in 2016. Perennial ryegrass (*Lolium perenne* L., cultivar ‘Linn’), Kentucky bluegrass (*Poa pratensis* L., cultivar ‘Ginger’), and tall fescue (*Schedonorus arundinaceus* (Schreb.) Dumort., cultivar ‘Lacefield Max QII™’) samples were from plots established in 2018. Samples (500 g fresh weight) were cut 5 cm above the base, and seed heads and dead grass blades were removed. Samples were stored on ice during transport, frozen, and lyophilized (Botanique Model 18DX48SA freeze-dryer). Dried samples were milled (<0.5 mm) using a Thomas Scientific Model 4 Wiley mill.

#### 2.1.2 Enzymes

Endoxylanase enzymes (E-XYACJ, GH10 from *Cellvibrio japonicus*, and E-XYLNP, GH11 from *Neocallimastix patriciarum*) were purchased from Megazyme (Bray, County Wicklow, Ireland). Starch-degrading enzymes (thermostable alpha-amylase, Termamyl SC^®^; and amyloglucosidase, AMG 300^®^) were from Novozymes (Bagsværd, Denmark).

#### 2.1.3 Chemicals and reagents

High-purity (≥99.95%) acetanilide was from Sigma-Aldrich. Deuterium oxide D_2_O (NMR solvent, purity 99.9%) was from Magni-Solv (EMD Millipore). Lactose monohydrate (≥99.5%) was from Sigma-Aldrich. Sodium hydroxide solution (50% w/w solution) and anhydrous sodium acetate (NaOAc) for preparing HPAEC eluent were from Fisher Chemical and OmniPur (EMD Millipore), respectively. Trifluoroacetic acid (TFA) and selected phenolic acid standard compounds (*trans-*caffeic, *ortho-*coumaric, *trans-para-*coumaric, *trans-*ferulic, isoferulic, and *trans-*sinapic) were obtained from VWR. *Cis*-ferulic acid and *cis*-*para-*coumaric acid were prepared by exposing a stock solution containing *trans*-ferulic acid and *trans*-*para-*coumaric acid (5 mM each, prepared in 50/50 MeOH/H_2_O, v/v) to UV radiation (366 nm) overnight. All water for analyses and HPAEC eluents was deionized and then further purified (resistivity ≥18.2 MΩ-cm) using a Barnstead Nanopure Diamond purification system.

#### 2.1.4 Oligosaccharide standard compounds

**Figure 1:**
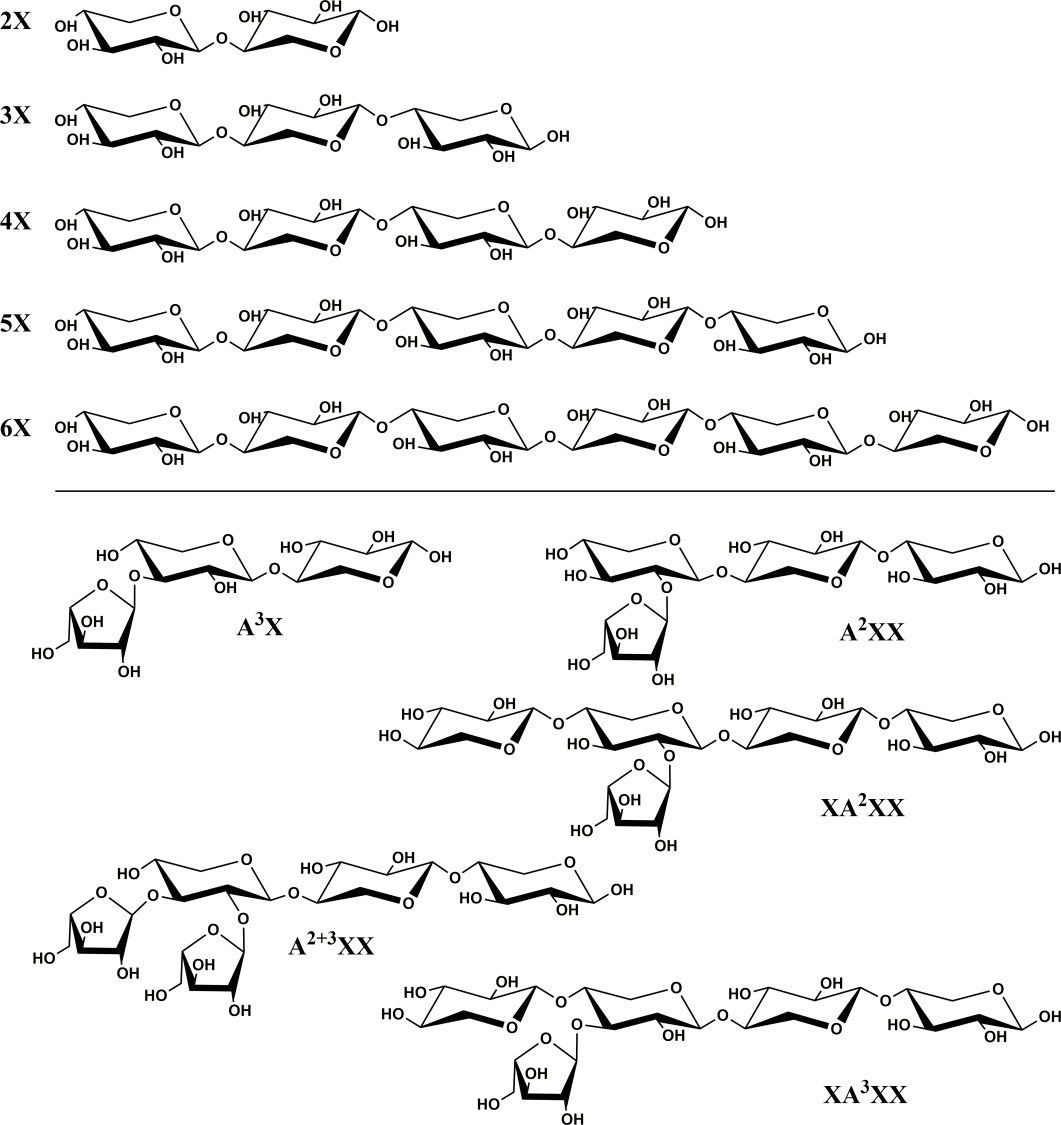
Chemical structures and abbreviations of oligosaccharide standard compounds utilized in study. Branched oligosaccharides were named following the system proposed by Fauré et al. (2009).

Xylooligosaccharide [XOS; degree of polymerization (DP) 2-6; obtained as individual compounds] and arabinoxylan oligosaccharide (AXOS) standard compounds were purchased from Megazyme [see **Figure 1** for structures and abbreviations; branched oligosaccharides were abbreviated following the system proposed by Fauré et al. (2009)]. Stock solutions of the compounds were prepared, and, due to the hygroscopicity of these materials, the exact concentration of the stock solutions was determined *via* quantitative ^1^H-NMR using the method described by Rundlöf et al. (2014) with high-purity acetanilide as the weighing-in internal standard.

### 2.2 Preparation and characterization of water-insoluble plant cell wall material from grasses

#### 2.2.1 Preparation of water-insoluble plant cell wall material from cool-season grass species

Lyophilized, milled grass material (4 g per replicate, with four replicates per species) was suspended in water and stirred at ambient temperature for 1 h. The slurry was centrifuged, and the supernatant was discarded to remove water-soluble carbohydrates. The pellet was transferred to an Erlenmeyer flask (200 mL), phosphate buffer (50 mL, 0.08 M, pH 6.2) and alpha-amylase (300 µL) were added to the pellet, and the mixture was incubated for 20 min at 92°C in a shaking water bath. The samples were cooled to room temperature over ice, and the pH was adjusted to 4.5 using 0.5 M HCl. Amyloglucosidase (150 µL) was added, and samples were incubated for 30 min in a stationary water bath at 60°C (with swirling every 5 min). Samples were centrifuged (15 min, 5360 *g*), the supernatant was carefully removed, and the pellet was washed twice with warm water (60°C, 1×100 mL and 1×50 mL), three times with absolute ethanol (2×100 mL and 1×50 mL), and three times with acetone (2×100 mL and 1×50 mL), while centrifuging and discarding the supernatant between each step. Acetone was volatilized from the washed residue in a fume hood, then the residue was dried in a vacuum drying oven (90 mbar, 70°C, 22 h), and stored in a desiccator until analysis.

#### 2.2.2 Determination of monosaccharide profile of grass cell wall material

Dried water-insoluble grass cell wall material (100 mg) was weighed into a 50-mL glass Pyrex tube along with glass beads, 1.5 mL of 12 M H_2_SO_4_ was added, and the slurry was vortexed for 1 min. Samples were put on ice for 30 min, vortexing for 1 min at 10-min intervals. Samples were allowed to stand at room temperature for an additional 2 h, vortexing for 1 min at 30-min intervals. Each sample was diluted with water (9.75 mL), vortexed for 1 min, and placed in a heating chamber (100°C) for 3 h. After cooling, the sample was filtered through a PTFE syringe filter (0.2 µm pore size), and a 5-mL aliquot of filtrate was neutralized with 4 M NaOH (4.3 mL, add dropwise until pH range between 5-7 is reached), and then brought up to a final volume of 50 mL in a volumetric flask with deionized water. Samples were diluted 1:10 prior to HPAEC-PAD injection. Diluted samples were separated on a CarboPac PA-1 anion-exchange column (250 × 4 mm; Thermo Scientific Dionex) preceded by a guard column (4 × 50 mm) in an ICS-5000+ HPAEC-PAD system from Thermo Scientific Dionex equipped with an AS-AP autosampler, dual pump, and DC electrochemical detector. The injection volume was 25 µL, and the flow rate was 1 mL/min with a ternary gradient (eluent A: deionized water; eluent B: 0.1 M NaOH stored under a headspace blanket of nitrogen gas; eluent C: 0.2 M NaOAc in 0.1 M NaOH and stored under a headspace blanket of nitrogen gas; gradient conditions at injection 90% A, 10% B, 0% C; linear from 0-1.5 min following sample injection to 96% A, 4% B, 0% C; hold until 25 min; linear from 25-35 min to 0% A, 100% B, 0% C; abrupt change to 0% A, 0% B, 100% C; hold from 35-45 min; abrupt change to 0% A, 100% B, 0% C; hold from 45-55 min; abrupt change to 90% A, 10% B, 0% C; hold from 55-65 min). A quadruple potential detector waveform was implemented [“carbohydrate (standard quad)”] with a gold working electrode, and both the column and detector compartments were heated to 30°C. Five-point standard calibration curves were created from monosaccharide standard compounds for two concentration ranges (1-25 µM and 25-125 µM) for the main sugars released in the grass samples by hydrolysis (rhamnose, arabinose, galactose, glucose, and xylose). The resulting chromatographic data were analyzed using the Chromeleon software program (Thermo Scientific Dionex), and peak areas were fitted to quadratic curves in OriginPro 2017. The complete analysis, hydrolysis through chromatography, was performed on four biological replicates for each grass species.

#### 2.2.3 Determination of ester-linked phenolic acid content of grass cell wall material

Dried water-insoluble grass cell wall material (100 mg) was weighed into a 50-mL glass Pyrex tube, 5 mL of 2 M NaOH was added, and the sample was vortexed for 1 min. Internal standard (50 µL of 5 mM *trans-o-*coumaric acid, for a total of 0.25 µmol) and a small magnetic stir bar were added to each tube, and the samples were capped and allowed to hydrolyze at room temperature in the dark for 18 h with constant stirring. Samples were acidified to pH<2 with 2 mL of 12 N HCl, and the protonated phenolic acids were partitioned three times with diethyl ether (6 mL, 5 mL, 5 mL). After each addition of diethyl ether, samples were centrifuged to separate the organic and aqueous layers, and the separated organic layers were collected and combined for each replicate. Ether extracts were evaporated to dryness under a stream of nitrogen gas. Dried residues were dissolved in 1 mL MeOH/H_2_O (50/50 v/v), leading to an internal standard concentration of 250 µM *trans-o-*coumaric acid. Samples were subsequently diluted 1:10 in 250 µM *ortho*-coumaric acid solution prepared in MeOH/H_2_O (50/50 v/v) prior to HPLC analysis with diode-array detection (DAD) using a Shimadzu 20-AR system equipped with a SIL-20AHT autosampler, two LC-20AT pumps, and a SPD-M20A photodiode array detector. Samples (10 µL injection volume) were separated on a Phenomenex Luna phenyl-hexyl column (250 × 4.6 mm, 5 µm particle size) using the following binary gradient: eluent A = 1 mM TFA; eluent B = [90/10 v/v (acetonitrile)/(1 mM TFA in 50/50 v/v MeOH/H_2_O)]; gradient condition at injection 88% A, 12% B; hold for 13 min; linear from 13-23 min from 12 to 15% B; hold from 23-28 min, linear from 28-33 min from 15 to 16% B; linear from 33-37 min from 16 to 66% B, hold from 37-42 min, linear from 42-43 min back to starting conditions of 88% A, 12% B; with re-equilibration for 10 min. Compounds were detected at 325 nm and quantified with linear, equidistant, 6-point internal calibration curves (*trans-*ferulic and *trans-p-*coumaric acid, 100-1000 µM; *cis-*ferulic and *cis-p-*coumaric acid, 10-100 µM and 7-70 µM, respectively), using *ortho*-coumaric acid as the internal standard (250 µM). The complete analysis, hydrolysis through chromatography, was performed on at least three biological replicates for each species.

### 2.3 HPAEC-PAD-based quantification of endoxylanase-released xylooligosaccharides and arabinoxylan oligosaccharides from grass arabinoxylans

#### 2.3.1 HPAEC method optimization for oligosaccharides

An HPAEC gradient was optimized which enabled consistent separation of the ten XOS and AXOS standard compounds and the selected internal standard, lactose. Samples (25 µL injection volume) were separated on a CarboPac PA-200 column (3 × 250 mm) equipped with a guard column (3 × 50 mm) at 30°C. The flow rate was 0.4 mL/min with a ternary gradient (eluent A: deionized water; eluent B: 0.1 M NaOH stored under a headspace blanket of nitrogen gas; eluent C: 1 M NaOAc in 0.1 M NaOH and stored under a headspace blanket of nitrogen gas; gradient conditions at injection 75% A, 25% B, 0% C; linear from 0-10 min to 100% B; hold from 10-20 min; linear from 20-24.5 min to 97.5% B, 2.5% C; hold from 24.5-29.5 min; linear from 29.5-55 min to 83.4% B, 16% C; abrupt change to 100% C; hold from 55-75 min; abrupt change to 100% B; hold from 75-95 min; abrupt change to 75% A, 25% B; and re-equilibrate for 10 min). The “carbohydrate (standard quad)” quadruple potential detector waveform was used with a gold working electrode.

#### 2.3.2 Validation of HPAEC quantification method

All validation steps of the HPAEC quantification method were performed using the optimized gradient. Lactose (0.5 µM) was incorporated into all samples as the internal standard. A reliable working concentration range (CR) was assessed by preparing standard solutions in triplicate, then preparing and injecting at least seven equidistantly-spaced sample concentrations. Both a high-range (approximately 0.3 – 3 µM; five concentration points) and low-range (approximately 0.03 – 0.3 µM; five concentration points) standard quantification curve were prepared within the CR for each oligosaccharide. Peak areas were integrated using Chromeleon software, and quadratic standard curves were calculated with OriginPro 2017 software using [(peak area oligosaccharide)/(peak area lactose internal standard)] as the dependent (y) variable and the oligosaccharide concentration as the independent (x) variable. Additionally, the relative response factor (RRF) for all standards against lactose was calculated using the CR data set. The [(AXOS concentration) × (lactose peak area)] values were plotted as the independent variables, and the [(lactose concentration) × (AXOS peak area)] values were plotted as the dependent variables. The slopes of the resulting linear regression lines were the RRF values of the individual standard compounds compared to lactose on a molar basis.

The limit of detection (LOD) and limit of quantification (LOQ) for each compound were determined by preparing a 5-point standard curve in triplicate at a very low, but detectable, concentration range (0.01-0.1 µM). This concentration range was determined based on the minimal visual distinction of analyte from baseline noise. Peak areas were determined, and linear regression analysis was performed in Excel to determine the standard error of the regression line (SER) and the regression slope (RS). LOD and LOQ were calculated using the following equations (Eq. 1 and 2, respectively) from the International Council for Harmonisation of Technical Requirements for Pharmaceuticals for Human Use guidelines (Ermer and Miller, 2006):


(1) $$LOD=\frac{3.3\;SER}{RS}$$

(2) $$LOQ=\frac{10\;SER}{RS}$$

#### 2.3.3 Method application to forage cell walls

A working enzyme solution (12.5 U/mL) of *Cellvibrio japonicus* was freshly prepared in water. Insoluble grass cell wall material (30 mg) was weighed into a 2-mL Eppendorf tube with a screw cap, 24 µL of working enzyme solution and 1176 µL of water were added, and samples were incubated in a thermoshaker dry bath (Grant Instruments PHMT-PSC24) for 12 h at 60°C and 600 rpm. Incubation was stopped by placing samples in a hot water bath (95°C) for 15 min to deactivate enzymes. Samples were centrifuged at 16,000 *g* for 10 min. An aliquot (500 µL) of supernatant was removed, mixed with 1 mL of water containing the lactose internal standard to dilute (final lactose concentration = 0.5 µM), filtered through a PTFE syringe filter (0.22 µM pore size), and analyzed *via* the optimized HPAEC-PAD method. Released oligosaccharides were identified by comparing peak retention times with the authentic standard compounds and quantified using quadratic calibration curves. A new set of calibration curves was measured on the HPAEC-PAD with each new batch of eluent.

### 2.4 Statistical analysis

Effects of species differences in molar proportions of cell wall monosaccharides, concentrations of ester-linked phenolic acids, and concentrations of xylose and individual oligosaccharides following endoxylanase digestion were determined by analysis of variance (ANOVA) using a 1-way model. Data were tested for normality *via* the Kolmogorov-Smirnov test. If significant differences (*p<*.05) were seen, means were compared using a Dunn-Sidak (cell wall monosaccharides and ester-linked phenolic acids) or Bonferroni (oligosaccharides) test. All statistical analyses were completed using the OriginPro 2017 software program from OriginLab Corporation.

## 3 Results and discussion

### 3.1 Monosaccharide composition of the water-insoluble plant cell wall material of cool-season forages

The monosaccharide composition of the water-insoluble plant cell wall material was quantified *via* HPAEC-PAD following Saeman hydrolysis (see **Table 1**). Glucose, xylose, arabinose, galactose and rhamnose were quantified in all pasture grass samples (with the exception of rhamnose in perennial rye). The concentration of glucose in the cell wall was significantly lower in perennial ryegrass (45% of the monosaccharides released during hydrolysis) than for the other three species (52-57% of the monosaccharides released during hydrolysis), indicating smaller amounts of cellulose in the cell walls of perennial ryegrass. The proportion of glucose in the sugars released during hydrolysis of our perennial ryegrass cell wall material (45%) was also lower than the range (50-60%) reported by Gordon et al. (1985) for epidermis, mesophyll, and secondary cell walls of perennial ryegrass. Galactose levels, which could arise from complex, galactose-bearing side-chains in the AX (Schendel et al., 2016), were low overall (2-3% of the released monosaccharides), but were significantly higher in tall fescue and bluegrass compared to perennial rye and timothy. Galactose could also partially originate from rhamnogalacturonan I pectic polysaccharides (galactan side chains), which are observed at low levels in the primary cell wall of grasses (Chesson et al., 1995; Xue et al., 2013; Couture et al., 2021; Avci, 2023). Trace amounts of rhamnose, which also arise from rhamnogalacturonans, were seen in all samples. The arabinose/xylose (A/X) ratios of all samples were quite low (see **Table 1**), ranging from 0.22 to 0.29. Perennial rye had a significantly lower A/X ratio compared to bluegrass and timothy, but not when compared to tall fescue. These values indicate a low level of xylan backbone substitution and are comparable to those previously reported in the literature. Gordon et al. (1985) described A/X ratios for perennial ryegrass leaves of 0.66, 0.40, and 0.14 for mesophyll cell walls, epidermis, and the residual fiber fraction, respectively. Sijtsma and Tan (1993) and Meak (2002) reported A/X ratios of 0.36 and 0.26, respectively, for perennial ryegrass cell walls isolated from whole plants, and Xu et al. (2007) found A/X ratios ranging from 0.25 to 0.45 for several water-insoluble AX fractions from perennial ryegrass leaves. Collings and Yokoyama (1979), Åman and Lindgren (1983), Meak (2002), and Kasuya et al. (2008) reported A/X ratios of 0.24, 0.21, 0.23, and 0.25 respectively, for tall fescue. Lindgren et al. (1980) reported A/X ratios for timothy grass throughout the growing season ranging from 0.21 to 0.32. Meak (2002) found a similar ratio of 0.25 for timothy grass. A/X ratios of 0.25 and 0.29 were respectively described for Kentucky bluegrass by Collings and Yokoyama (1979) and Meak (2002).

**Table 1:** Neutral monosaccharide composition of water-insoluble cell wall material isolated from leaf tissue of cool-season forage species.

Forage species	Rhamnose, mg/g		Arabinose, mg/g		Xylose, mg/g		Glucose, mg/g		Galactose, mg/g		A/X
	mean	SD	mean	SD	mean	SD	mean	SD	mean	SD	
*Kentucky bluegrass*	3.4^a^	0.27	53.3^a^	2.7	185.8^a^	8.2	312.0^a^	16.1	20.6^a^	0.85	0.29^a,b^
*Tall fescue*	3.4^a^	0.10	49.1^a^	1.7	190.4^a^	2.0	293.4^a^	2.9	20.2^a^	1.0	0.26^b,c^
*Perennial ryegrass*	trace	–	45.4^a^	4.4	203.1^b^	4.8	216.9^b^	7.5	14.6^b^	1.7	0.22^c^
*Timothy*	9.9^b^	0.87	51.0^a^	2.4	193.4^a^	12.1	356.1^a^	14.2	17.1^b^	0.85	0.26^a,b^

A/X, arabinose/xylose ratio; SD, standard deviation.

Values represent released monosaccharides (mg) per gram of water-insoluble cell wall material. Means and SD were calculated from 4 replicate determinations from each species. Means with different letters in the same column are statistically different between species (p< 0.05); means with shared letters in the same column are statistically equivalent.

### 3.2 Ester-linked phenolic acid content of the water-insoluble plant cell wall material of cool-season forages

The insoluble cell wall material of four grass samples contained ester-linked *p*-coumaric and ferulic acid in both their *trans-* and *cis-* stereoisomers (see **Supplementary Figure 1** for sample chromatogram). The total levels of monomeric ester-linked phenolic acids were over 10,000 µg g^-1^ water-insoluble cell wall material (**Table 2**), representing a full one percent of the insoluble material. The *cis* isomers of ferulic and coumaric acid arise from UV light-induced isomerization (Kahnt, 1967), so although plants synthesize these compounds exclusively in their *trans-* forms, formation of the *cis-*isomers is to be expected in forage tissues exposed to sunlight (Yamamoto and Towers, 1985; Turner et al., 1993; Schäfer et al., 2019). Timothy had significantly lower concentrations of *p-*coumaric acid than the other species. *Trans*-ferulic acid concentrations were not significantly different between the pasture grasses and were roughly comparable to the *trans-p-*coumaric acid concentrations. Approximately equivalent amounts of ester-linked ferulates and coumarates were also previously observed in aerial parts of timothy (Theander et al., 1981), ensiled tall fescue (Sousa et al., 2021), and aerial parts of rice (*Oryza sativa*) plants (Dhakarey et al., 2017). The roughly equivalent proportions of ester-linked ferulates and coumarates in vegetative tissue contrast with the ester-linked phenolic acid profiles of monocotyledonous grain tissue, which are dominated by ferulic acid (Andersson et al., 2008; Li et al., 2008; Nyström et al., 2008; Shewry et al., 2008; Schendel et al., 2015). In their ester-linked form, the two compounds are directly bonded to separate cell wall polymers: ferulic acid to AX and *p-*coumaric acid primarily to lignin (Smith and Hartley, 1983; Lu and Ralph, 1999; Hatfield et al., 2008), although small amounts of *p-*coumaric acid esters acylate AX (Mueller-Harvey et al., 1986). The clear difference in ester-linked phenolics between grain and vegetative tissue springs from their polymer composition: whereas vegetative tissue contains substantial amounts of lignin (Buxton and Russell, 1988), the lignin content of grain tissue is quite low (Bunzel et al., 2011).

**Table 2:** Monomeric ester-linked phenolic acids in water-insoluble cell wall material isolated from leaf tissue of cool-season forage species.

Forage species	*trans-p-*coumaric acid µg/g		*cis-p-*coumaric acid µg/g		*trans-*ferulic acid µg/g		*cis-*ferulic acid µg/g	
	mean	SD	mean	SD	mean	SD	mean	SD
*Kentucky bluegrass*	4384^a^	125	1094^a^	78	4143^a^	261	871^a^	43
*Tall fescue*	4279^a,b^	66	941^a^	32	4926^a^	242	851^a^	37
*Perennial ryegrass*	3581^b,c^	411	638^b^	124	5301^a^	586	791^a^	145
*Timothy*	2908^c^	357	389^c^	95	4804^a^	875	539^b^	98

SD, standard deviation.

Values represent released ester-linked phenolic acids (µg) per gram of water-insoluble cell wall material. Means and SD were calculated from 3 replicate determinations each for timothy and tall fescue and 4 replicates each for Kentucky bluegrass and perennial ryegrass. Means with different letters in the same column are statistically different between species (p< 0.05); means with shared letters in the same column are statistically equivalent.

We did not quantify dehydrodimers and higher oligomers of ferulates, representing AX-AX cross-links (Ralph et al., 1994; Bunzel, 2012), or ether-linked ferulates, representing AX-lignin cross-links (Quideau and Ralph, 1997). Ferulate-driven crosslinking significantly reduces AX degradability by endoxylanases (Grabber et al., 1998; Beaugrand et al., 2004; de Souza et al., 2018). Supplementation of xylanase-based enzyme treatments with feruloyl esterases increases the amount of released carbohydrate from AX in some cases (Li et al., 2011; Várnai et al., 2014), and some ruminant bacteria even express bifunctional endo-1,4-β-xylanase/esterases (Pavarina et al., 2021), thus directly attacking the ferulate-driven recalcitrance to xylanase digestion. However, although ester-linked ferulate monomers are relevant to cell wall architecture as potential building blocks for future cell wall polymer crosslinking, ferulate monomers themselves do not reduce xylanase-mediated hydrolysis of AX beyond the level caused by simple arabinosylation alone of the AX backbone (Grabber et al., 1998; Beaugrand et al., 2004; Várnai et al., 2014).

### 3.3 Optimization of endoxylanase digestion for production of oligosaccharide profiles in forage materials

Generation of oligosaccharide profiles by endoxylanase digestion followed by separation, detection, and (sometimes) quantification *via* HPAEC-PAD has been previously used to compare the fine AX structures of cereal grains. This approach generates and compares unique AXOS fingerprints for different grain varieties and grain tissues (Viëtor et al., 1994; Ordaz-Ortiz et al., 2005; Pastell et al., 2009; Saulnier and Quemener, 2009; Makaravicius et al., 2012; Snelders et al., 2013; Falck et al., 2014; Immerzeel et al., 2014). We have adapted this technique to fingerprint the AX contained in the vegetative tissue of cool-season forages. This method, which targets the water-insoluble hemicellulosic fraction of cool-season forages, complements the use of HPAEC-PAD to profile the water-soluble and ethanol-soluble carbohydrate fractions of various cool-season forages (Kagan et al., 2011; Kagan et al., 2018).

Hydrolysis of the β-1→4-glycosidic linkages between the xylopyranose units of the AX backbone by endoxylanases is the cornerstone of our approach, and which bonds are accessible by the enzymes is influenced by nearby side-chain residues and xylanase type (Pollet et al., 2010). Xylanases from the glycoside hydrolase (GH) family 10 require only two unsubstituted xylose units between branched units, whereas GH11 xylanases require three unsubstituted backbone units. The two enzyme types would thus produce different oligosaccharide fingerprints. GH10 xylanases are more effective in digesting more densely substituted AX, but the low A/X ratio of our vegetative grass tissue led us to expect that GH11 xylanases would still be able to access the xylan backbone. We compared a GH10 xylanase derived from *Cellvibrio japonicus*, a gram-negative soil bacterium (DeBoy et al., 2008), and a GH11 xylanase from *Neocallimastix patriciarum*, an anaerobic fungus isolated from ruminants (Orpin and Munn, 1986). Pure water was used as the incubation medium instead of buffer to avoid potential chromatography problems during HPAEC analysis. These conditions resulted in very limited oligosaccharide production by the GH11 *Neocallimastix patriciarum* enzyme (see **Supplementary Figure 2**), and therefore, only the *Cellvibrio japonicus* endoxylanase was used for further enzymatic work.

Enzyme incubation time was compared at 4, 12, 24, and 48 h. The 4 h incubation time resulted in only minimal hydrolysis, whereas by 12 h, release of various XOS and AXOS was clearly evident by examination of the HPAEC chromatogram and comparison with a standard chromatogram (data not shown). At 12 h, all of the DP 2-6 XOS were present, but at the later incubation times (24 and 48 h), the larger XOS had been mostly degraded into xylose and 2X. This was expected, given the hydrolytic patterns of GH10 endoxylanases. As reviewed by Pollet et al. (2010), 2X is the smallest XOS released by GH10 xylanases. We selected the 12 h incubation time as the standard condition for our forage AX fingerprinting method so that differences in the amount of unbranched sections along the AX backbone between materials would be more visible, and the AX fingerprint generated by the method would contain more structural information. Higher released quantities of longer XOS directly demonstrate the existence of many unsubstituted sections on the original AX polymer. In contrast, higher released quantities of 2X are less meaningful, since 2X could arise from degradation of a long stretch of unsubstituted xylan backbone, but it could also have been released from a region with a moderate degree of substitution.

### 3.4 Validation of HPAEC-PAD-based quantification method for XOS and AXOS

**Figure 2:**
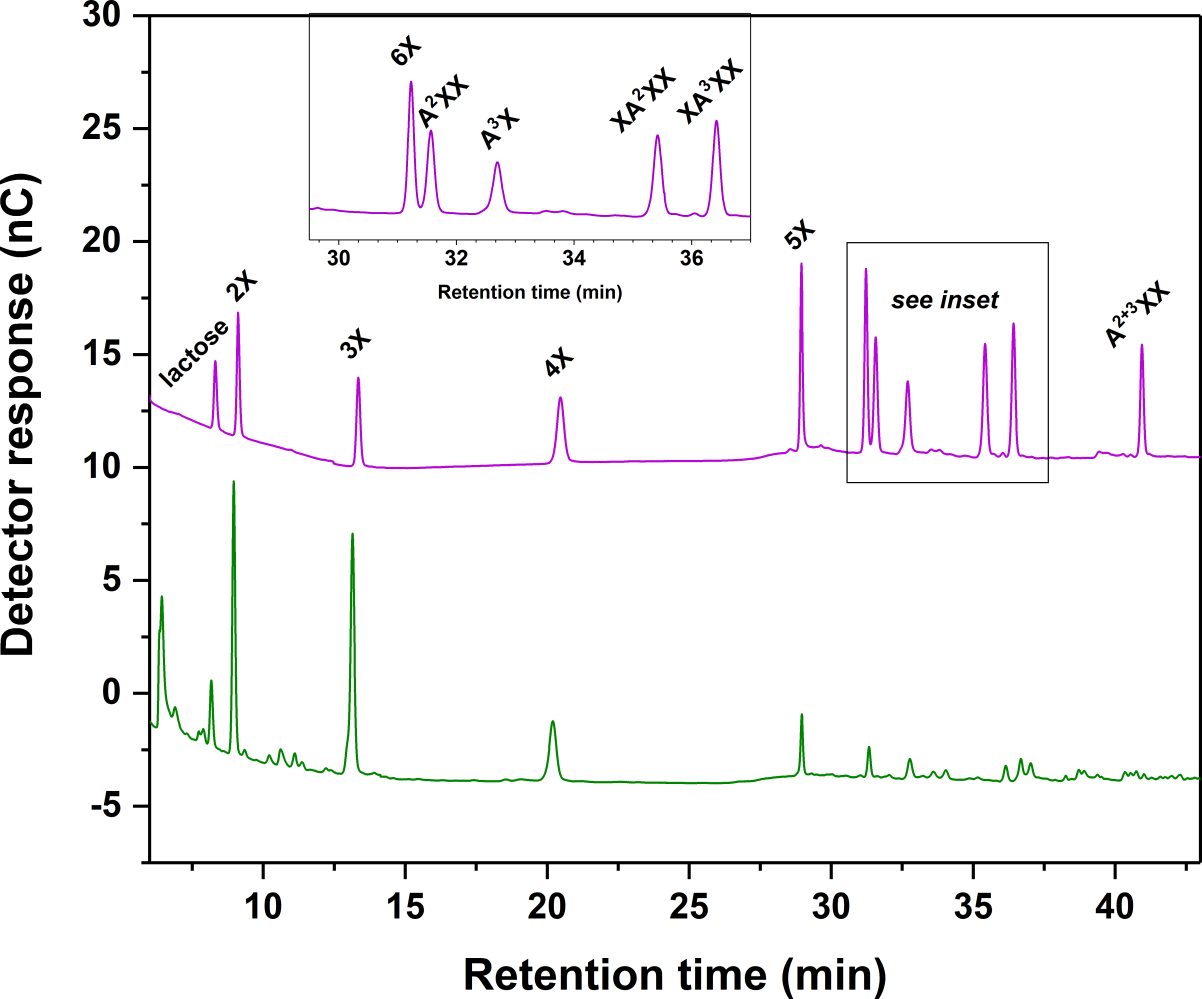
Chromatographic separation of linear xylooligosaccharide (XOS) and branched arabinoxylan oligosaccharide (AXOS) compounds using high-performance anion-exchange chromatography with pulsed amperometric detection (HPAEC-PAD). Upper chromatogram and inset: XOS and AXOS standards and lactose internal standard. Lower chromatogram: Endoxylanase hydrolysate of insoluble cell wall material from timothy grass (*Phleum pratense* L., cultivar ‘Clair’) vegetative tissue. Please refer to **Figure 1** for chemical structures corresponding to standard compounds’ abbreviations.

We selected 10 XOS and AXOS standard compounds (purchased commercially) and used quantitative ^1^H-NMR to weigh them in accurately. We chose lactose as an internal standard because it was well-separated from our standard compounds and not native to plant materials. Our optimized gradient and column temperature achieved baseline separation of the standard compounds and internal standard, with the exception of 6X and A^2^XX, which remained slightly shouldered (see **Figure 2**). As also reported by Alyassin et al. (2020), implementation of a gradient elution step for sodium hydroxide at the beginning of each run as opposed to an isocratic level of sodium hydroxide substantially improved oligosaccharide resolution. We also found that a relatively high concentration of sodium acetate (1 M) during the column clean-out step followed by a 20-minute rinse with 0.1 M sodium hydroxide improved separation reproducibility. **Table 3** presents retention time (RT) values from a randomly selected standard chromatogram prepared using the optimized gradient. Intraday RT reproducibility was excellent (± 0.05 min); small (maximum of 0.3 min) interday variations in RT were observed between eluent batches, but because a fresh standard sample was run with each new batch of eluent, peak ID was unhindered.

**Table 3:** Method validation parameters for arabinoxylan oligosaccharide standard compounds.

Compound^*^	RT (min)	LOD (µM)	LOQ (µM)	RRF	CR^**^ (µM)	R^2^, linear fit	R^2^, quadratic fit	Quadratic calibration equation
***lactose (internal standard)***	8.291	n/a	n/a	1	n/a	n/a	n/a	n/a
***xylose***	5.802	0.005	0.016	0.571	0.03-6	0.9992	0.9997	y = 0.05128 + 1.23409x -0.01538x^2^
***2X***	9.106	0.007	0.021	0.575	0.03-6	0.9989	0.9996	y = -0.0159 + 1.25436x -0.01735x^2^
***3X***	13.411	0.006	0.019	0.482	0.03-3	0.9869	0.9982	y = -0.0025 + 1.30658x -0.11716x^2^
***4X***	20.840	0.006	0.017	0.686	0.03-6	0.9942	0.9995	y = (-4.8706E-5) + 1.64344x -0.05445x^2^
***5X***	29.207	0.007	0.020	0.712	0.03-6	0.9942	0.9989	y = -0.00138 + 1.7258x -0.05485x^2^
***6X***	31.539	0.007	0.020	0.6791	0.03-6	0.9938	0.9997	y = -0.01063 + 1.99645x - 0.0688x^2^
***A^2^XX***	31.969	0.007	0.021	0.643	0.03-6	0.9971	0.9993	y = 0.0036 + 1.49302x -0.03454x^2^
***A^3^X***	33.183	0.008	0.023	0.516	0.03-6	0.9964	0.9977	y = 0.01515 + 1.12241x -0.02084x^2^
***XA^2^XX***	35.870	0.008	0.023	0.727	0.03-6	0.9959	0.9994	y = -0.01051 + 1.65177x -0.04592x^2^
***XA^3^XX***	36.824	0.006	0.018	0.660	0.033-6.63	0.9964	0.9996	y = -0.01288 + 1.5776x -0.0389x^2^
***A^2+3^XX***	41.194	0.007	0.022	0.555	0.03-6	0.9966	0.9994	y = -0.03066 + 1.31827x -0.0336x^2^

^*^ Please refer to **Figure 1** for chemical structures corresponding to standard compounds’ abbreviations.

^**^Range corresponds to the tested working concentration range.

CR, Concentration range; LOD, limit of detection; LOQ, limit of quantification; RRF, relative response factor; RT, retention time.

**Figure 3:**
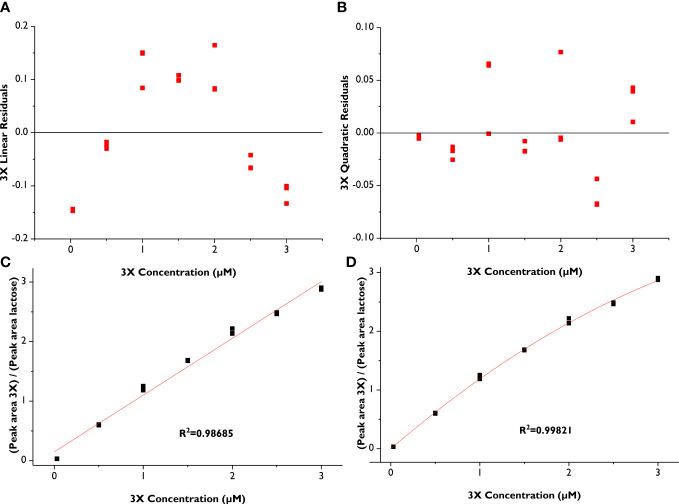
Comparison of linear vs. quadratic fit for xylotriose (3X) calibration curves (in triplicate). **(A)** Residual plot from linear fit; **(B)** Residual plot from quadratic fit, **(C)** Linear calibration curve; **(D)** Quadratic calibration curve.

The LOD and LOQ for xylose and the oligosaccharide standards ranged from 5 to 8 nM and 16 to 23 nM, respectively, underscoring the outstanding sensitivity of the amperometric detector (**Table 3**). Due to limited quantities of standard compounds, we did not attempt to identify a precise upper concentration limit of the compounds where detector saturation would hamper quantitative work, but instead determined a reliable working concentration range, that is, one above the LOQ and well below detector saturation (**Table 1**). Individual standard curves were prepared for each compound within the CR (**Table 1**). We compared fitting data to both linear and polynomial (quadratic) equations and found that, although the R^2^ values were high for the linear fit (≥0.986 for all standards), the R^2^-values for a quadratic fit were slightly higher for each standard (**Table 1**). Visual comparison of the residual plots from the linear vs. quadratic fit further supported the use of a quadratic equation: whereas the linear residual plots had a distinctive parabola pattern, the quadratic residual plots were randomly dispersed (**Figure 3** residual plots). Other researchers have also utilized quadratic calibration curves for HPAEC-PAD-based oligosaccharide quantification, for instance, for human milk oligosaccharides (Kunz et al., 2017), β-glucan-derived oligosaccharides (Radva et al., 2012), and fructans (Haselberger and Jacobs, 2016; Ispiryan et al., 2019). Alyassin et al. (2020) utilized linear calibration curves for HPAEC-PAD-based quantification of XOS and AXOS, but they did not compare quadratic fitting.

We also calculated RRF values for each compound (**Table 3**), but given the reported analyte-specific PAD response drop due to recession of the gold working electrode for XOS and AXOS (Mechelke et al., 2017), we chose to perform all oligosaccharide quantifications using quadratic calibration curves measured anew with each batch of eluent. The molar detector responses for xylose and 2X were similar to each other and higher than that of 3X, but the RRFs for the DP 4-6 XOS were higher than the lower-DP compounds. Increasing molar response values with increasing DP up to a DP 6-7, followed by a leveling of detector response, has been reported for some oligosaccharide series, including dextrins (White et al., 2003) and fructooligosaccharides (Fernández et al., 2013). In contrast, Mechelke et al. (2017) reported a decreased molar detector response for DP 4-5 XOS compared to DP 1-3, no clear trend for cellooligosaccharides, and decreasing molar detector responses for DP 2-5 laminarioligosaccharides.

### 3.5 Quantitative profiling of cool-season forage oligosaccharides released by *Cellvibrio japonicus* endoxylanases

**Figure 4:**
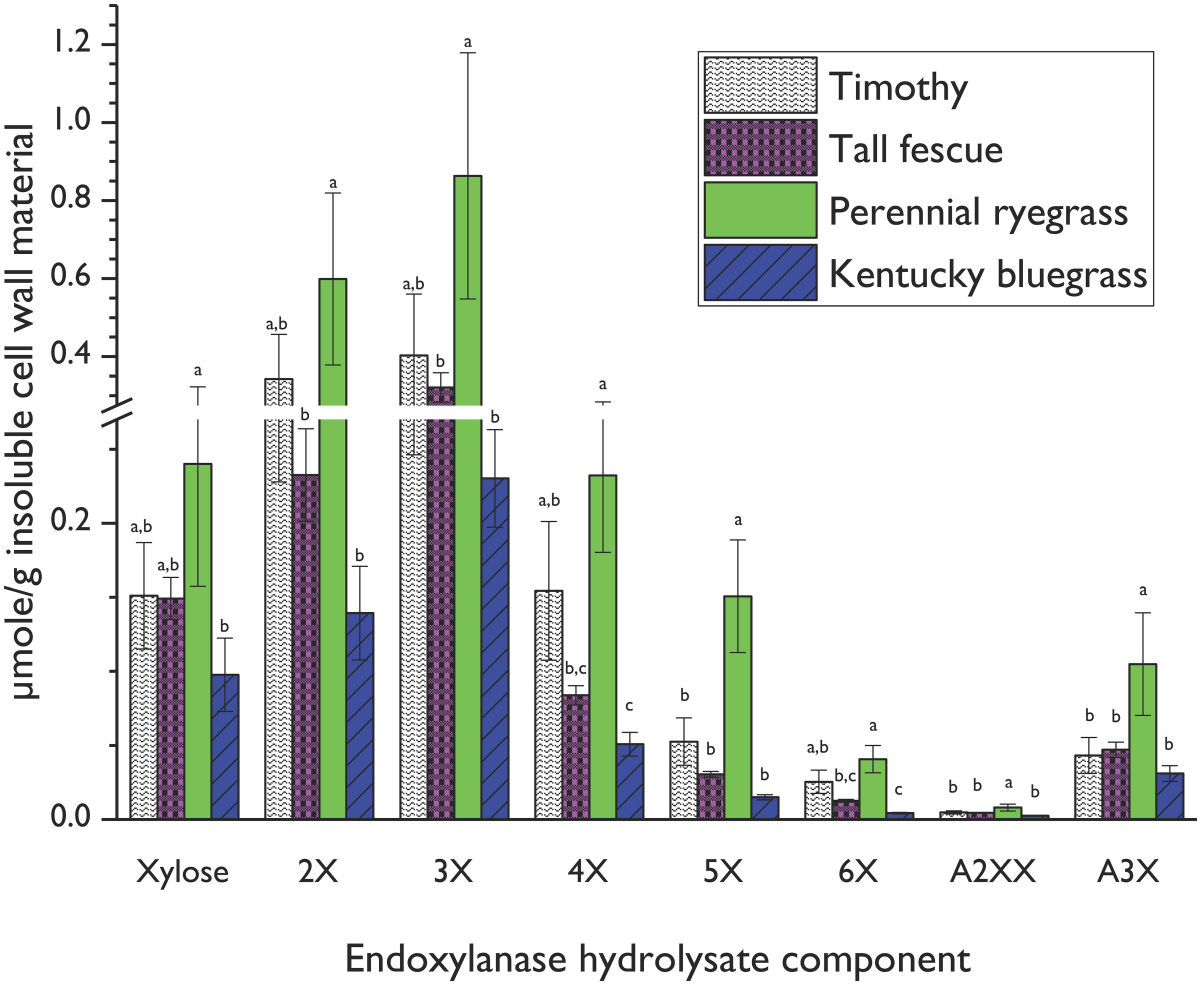
Xylooligosaccharide (XOS) and arabinoxylan oligosaccharide (AXOS) profiles of endoxylanase (*Cellvibrio japonicus*; GH 10) hydrolysates from timothy, tall fescue, perennial ryegrass and Kentucky bluegrass insoluble cell wall material. Data represent means and standard deviations of four biological replicates for all compounds, except for 3X from perennial rye grass and timothy and xylose from tall fescue (n=3 in these cases). Lowercase letters above error bars indicate results of statistical comparisons of the means between species for individual XOS and AXOS. When considering results for an individual XOS or AXOS, means lacking a common letter are different for that individual XOS or AXOS between species (*p*< 0.05). Please refer to **Figure 1** for chemical structures corresponding to standard compounds’ abbreviations.

The validated HPAEC-PAD method enabled quantitative comparisons of endoxylanase hydrolysates of cool-season forage samples (see **Figure 4**). Many previous studies of vegetative AX structures released by xylanase were based on alkali-pretreated material (Mazumder and York, 2010; Bowman et al., 2014; Tryfona et al., 2019), but because these treatments cleave ester linkages, AX-AX crosslinks are undone and AX is unzipped from lignin by releasing the ester bonds of ester/ether-linked ferulates, which dramatically alters the xylanase accessibility compared to the native cell wall structure. We chose to work with the native, non-pretreated structure to strengthen the practical application of our results to forage screening for livestock feeding. Only a small amount of AX was released from the native structure as AXOS during the 12 h of water-based endoxylanase incubation (0.09, 0.14, 0.19, and 0.39% of the total arabinose and xylose for Kentucky bluegrass, tall fescue, timothy, and perennial rye, respectively). Incubation in buffer would likely increase xylanase activity and AX solubilization, but the recommended buffer for most commercially available endoxylanases, sodium acetate, could influence HPAEC separation, so we chose to incubate in pure water instead. However, the intent of the AX profiling method was not full degradation of the cell wall AX of forage material into AXOS, but rather, the rapid generation of thumbnail sketches of the AX structure in forage materials. Differences in these thumbnail sketches, produced by digesting various forage materials under the same incubation conditions, serve as unique fingerprints of the AX contained in those forages and can be used to compare the AX structures contained in those forages.

Perennial rye generated significantly greater amounts of released xylose and all quantifiable oligosaccharides compared to bluegrass, and significantly greater amounts of 5X, A^2^XX, and A^3^X compared to timothy and tall fescue. Several competing AX structural factors influence endoxylanase hydrolysis efficiency in intact cell walls. Firstly, densely substituted AX backbone regions block endoxylanase access to the xylan backbone (Pollet et al., 2010) and thus require the deployment of accessory enzymes for complete depolymerization (de Vries et al., 2000). Secondly, denser AX backbone substitution patterns increase AX water solubility and limit hydrogen-bonding interactions with cellulose microfibrils (Kabel et al., 2007; Köhnke et al., 2008; Saulnier et al., 2012; de Souza et al., 2013; Selig et al., 2015). As discussed in Section 3.1, both the glucose content, corresponding to cellulose, and the A/X ratio, corresponding to AX backbone substitution, of perennial ryegrass cell wall material was lower than that of the other grass species. The higher levels of released XOS and AXOS from the perennial rye cell wall are thus partly due to the lower quantities of cellulose present in the perennial rye cell wall, which would reduce hydrogen bonding opportunities between the cellulose microfibrils and the AX, and the lower backbone substitution rate of the perennial rye AX polymer compared to the other forages.

Of the oligosaccharides and monosaccharides released, 3X was the most abundant across all grasses, but 4X, 5X, and 6X were also present in the hydrolysates from all species at the 12-h incubation point. This means that these species share a structural similarity of long stretches of unsubstituted regions on the AX backbone.

A^2^XX and A^3^X were the only quantifiable branched AXOS released under the current conditions. The smallest decorated oligosaccharide produced by a GH10 xylanase is A^3^X or A^2^X (Pell et al., 2004). Several small peaks that may have corresponded to XA^2^XX, XA^3^XX, or A^2+3^XX were seen in the chromatograms, but their identity and concentration could not be unequivocally determined because of partial coelution with other, unidentified peaks. Expansion of the method to include additional branched AXOS standards would improve the resolution of the generated AX fingerprints. Additional investigation with a mass spectrometer coupled to the HPAEC-PAD, analogous to the profiling method recently validated for enzymatic hydrolysates of xyloglucans (Steck et al., 2021), would strengthen our method by expanding our peak identification toolset beyond retention time comparison alone.

A peak that co-eluted with arabinose was seen in the monosaccharide region of the chromatogram for most, but not all, of the samples. However, xylanase-free blank incubations confirmed that the compound was released from the forage samples independently of the xylanase. As a result, we did not attempt to quantify any arabinose released during incubation, since our aim was quantification of the xylanase-generated AX profiles.

## 5 Conclusion

Monosaccharide composition of cell wall material provides a rudimentary view of the carbohydrate polymers, but more detailed structural information about the AX polymers in cool-season forages is lacking. A quantitative HPAEC-PAD method for xylanase-released AXOS was validated which permitted comparison of fine AX structures from forages. This method was applied to four cool-season pasture grass varieties after optimizing the enzymatic incubation process for the GH10 endoxylanase *Cellvibrio japonicus*. Perennial rye had a more abundant xylanase-mediated release of monosaccharides and oligosaccharides than timothy, tall fescue, and Kentucky bluegrass, which corroborated with perennial rye’s lower A/X ratio in its monosaccharide profile. 3X was the most abundantly-released oligosaccharide in all four grass species. Two branched AXOS were also successfully quantified. Ester-linked ferulic and coumaric acid monomers were quantified in both their *cis-* and *trans-*isomers in the four forages.

Future applications of the AX fingerprinting method include screening a wider pool of forage species and using the method to track and quantify forage AX fermentation by gut fermenting microorganisms. The HPAEC-based screening method could also be expanded to include other forage AX structural elements, such as substitution with glucuronic acid or 4-*O*-methylglucuronic acid.

## Data availability statement

The raw data supporting the conclusions of this article will be made available by the authors, without undue reservation.

## Author contributions

GJ: Sample and data analysis, data interpretation, and drafting of the manuscript. IK: Provided critical, constructive input throughout all stages of the study, significant contributions to HPAEC gradient development. MF and BD: Provided critical, constructive input throughout the study. RS: Conception of the study and experimental design (together with IK, MF, and BD), data analysis and interpretation, drafting of the manuscript. All authors have critically reviewed the manuscript and approved the publication of its content. All authors contributed to the article and approved the submitted version.
