# Supplementary material for: Profiling of cool-season forage arabinoxylans via a validated HPAEC-PAD method
Source: Front Plant Sci. 2023 Mar 13;14:1116995. doi: 10.3389/fpls.2023.1116995 (PMC10040848; doi:10.3389/fpls.2023.1116995)
Supplement: Supplementary file 1 [file DataSheet_1.pdf]

Supplementary Material

Profiling of Cool-Season Forage Arabinoxylans via a Validated HPAEC-PAD Method

Glenna E. Joyce[1], Isabelle A. Kagan[2], Michael D. Flythe[2], Brittany E. Davis[2], Rachel R. Schendel[1*]

[1]Department of Animal and Food Sciences, University of Kentucky, Lexington, KY 40546, USA

[2]Forage-Animal Production Research Unit, USDA-ARS, Lexington, KY 40546, USA

*** Correspondence:**
Dr. Rachel R. Schendel
rachel.schendel@uky.edu

1 Supplementary Data

1.1 Supplementary Figures

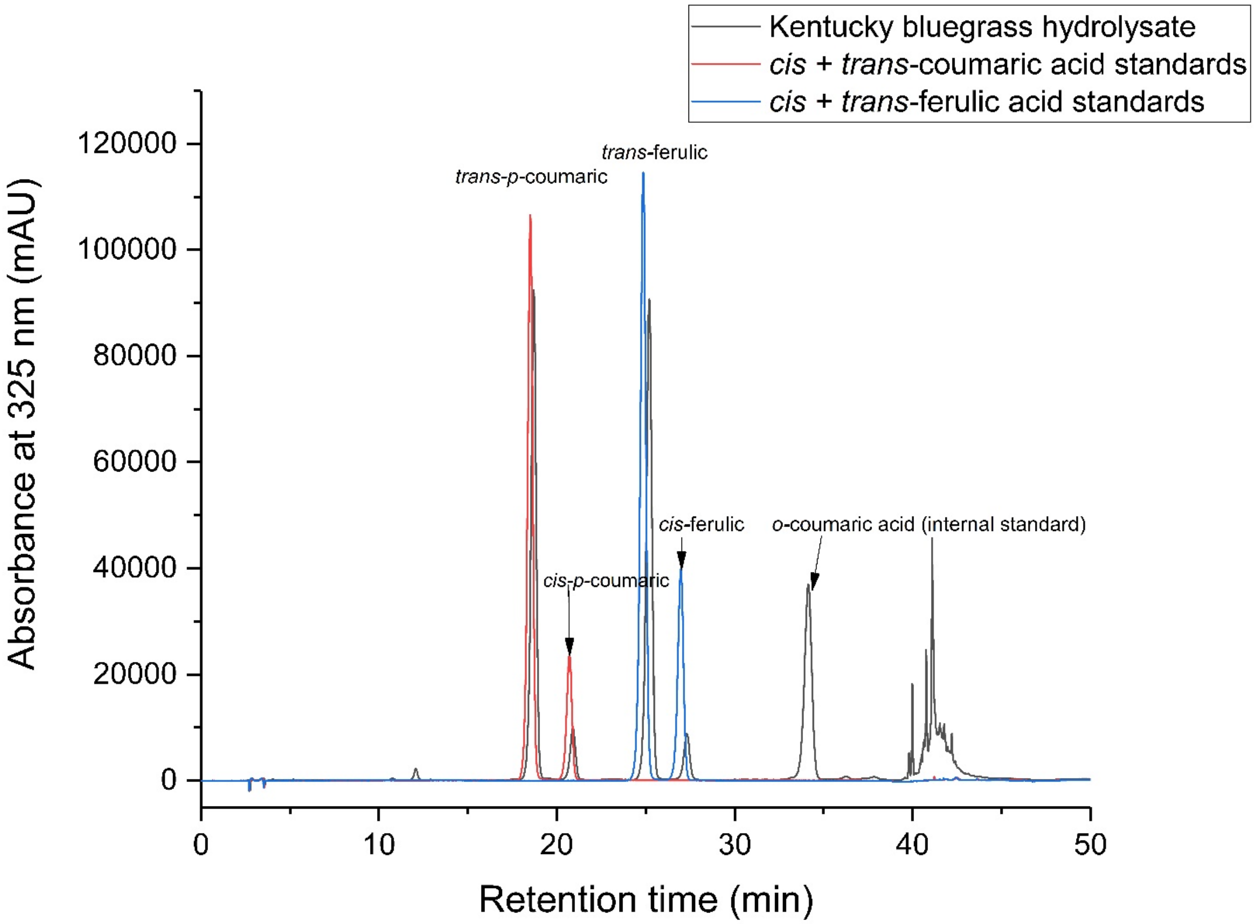

Supplementary Figure 1. Chromatograms showing separation of quantified phenolic acid compounds. Shown are a sample forage hydrolysate (Kentucky bluegrass, black trace), *trans*- and *cis*-*p*-coumaric acid standard compounds (red trace), and *trans*- and *cis*-ferulic acid standard compounds (blue).

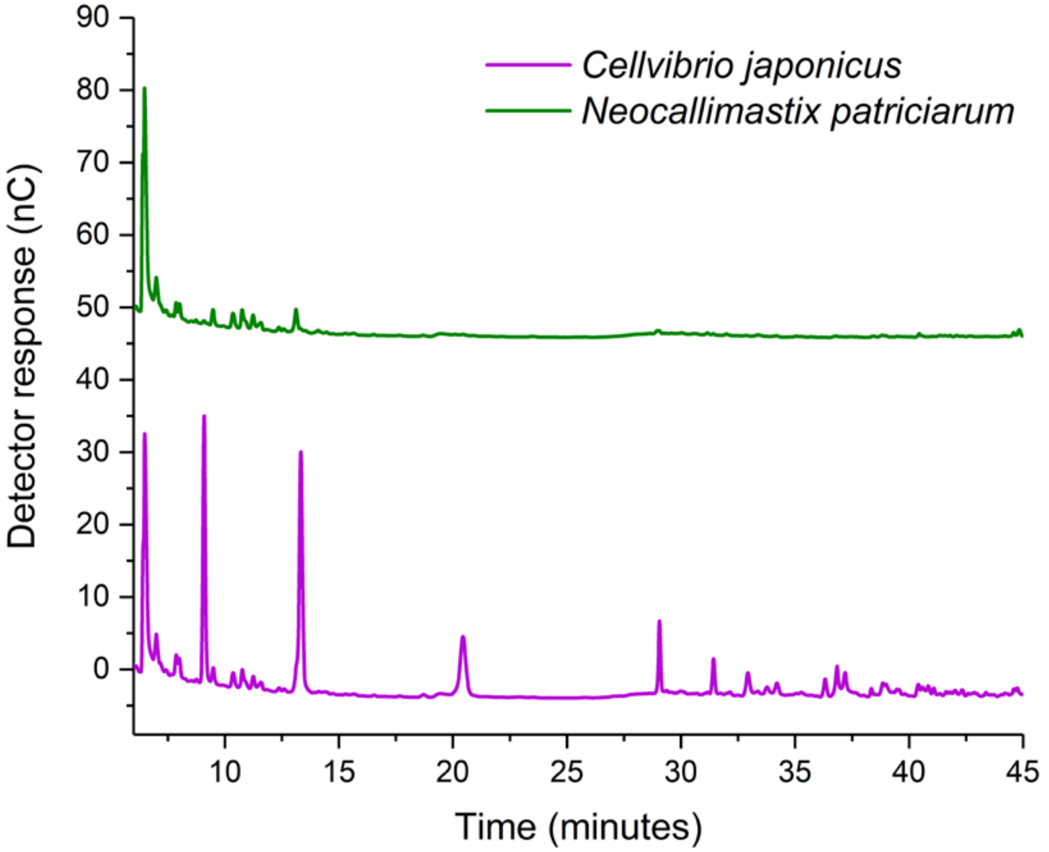

Supplementary Figure 2. Chromatograms comparing endoxylanase hydrolysates of insoluble cell walls of vegetative cool-season forage tissue by 12-hour incubation with *Cellvibrio japonicus* (CJ) GH10 endoxylanase (lower chromatogram) and *Neocallimastix patriciarum* (NP) GH11 endoxylanase (upper chromatogram).
